# Protocol and baseline data for a multi-year cohort study of the effects of different mass drug treatment approaches on functional morbidities from schistosomiasis in four African countries

**DOI:** 10.1186/s12879-017-2738-5

**Published:** 2017-09-29

**Authors:** Ye Shen, Charles H. King, Sue Binder, Feng Zhang, Christopher C. Whalen, W. Evan Secor, Susan P. Montgomery, Pauline N. M. Mwinzi, Annette Olsen, Pascal Magnussen, Safari Kinung’hi, Anna E. Phillips, Rassul Nalá, Josefo Ferro, H. Osvaldo Aurelio, Fiona Fleming, Amadou Garba, Amina Hamidou, Alan Fenwick, Carl H. Campbell, Daniel G. Colley

**Affiliations:** 1 Department of Epidemiology and Biostatistics, University of Georgia, Athens, GA USA; 2 Center for Global Health and Diseases, Case Western Reserve University, Cleveland, OH USA; 3 Schistosomiasis Consortium for Operational Research and Evaluation, Center for Tropical and Emerging Global Diseases and Department of Microbiology, University of Georgia, Athens, GA USA; 4 Parasitic Diseases Branch, Division of Parasitic Diseases and Malaria, Centers for Disease Control and Prevention, Atlanta, GA USA; 5 Centre for Global Health Research, Kenya Medical Research Institute, Kisumu, Kenya; 6 Parasitology and Aquatic Diseases, Faculty of Health and Medical Sciences, University of Copenhagen, Copenhagen, Denmark; 7 National Institute for Medical Research, Mwanza Research Centre, Mwanza, Tanzania; 8 Schistosomiasis Control Initiative, Department of Infectious Disease Epidemiology, Imperial College, London, UK; 9 Instituto Nacional de Saúde, Maputo, Mozambique; 10 Universidade Católica de Moçambique, Beira, Mozambique; 11 Réseau International Schistosomoses, Environnement, Aménagement et Lutte (RISEAL-Niger), Niamey, Niger

**Keywords:** Schistosomiasis, *Schistosoma haematobium*, *Schistosoma mansoni*, Morbidity, Drug therapy, Praziquantel, Africa, Cohort study, Kenya, Mozambique, Niger, Tanzania

## Abstract

**Background:**

The Schistosomiasis Consortium for Operational Research and Evaluation (SCORE) focus is on randomized trials of different approaches to mass drug administration (MDA) in endemic countries in Africa. Because their studies provided an opportunity to evaluate the effects of mass treatment on *Schistosoma*-associated morbidity, nested cohort studies were developed within SCORE’s intervention trials to monitor changes in a suite of schistosomiasis disease outcomes. This paper describes the process SCORE used to select markers for prospective monitoring and the baseline prevalence of these morbidities in four parallel cohort studies.

**Methods:**

In July 2009, SCORE hosted a discussion of the potential impact of MDA on morbidities due to *Schistosoma* infection that might be measured in the context of multi-year control. Candidate markers were reviewed and selected for study implementation. Baseline data were then collected from cohorts of children in four country studies: two in high endemic *S. mansoni* sites (Kenya and Tanzania), and two in high endemic *S. haematobium* sites (Niger and Mozambique), these cohorts to be followed prospectively over 5 years.

**Results:**

At baseline, 62% of children in the *S. mansoni* sites had detectable eggs in their stool, and 10% had heavy infections (≥ 400 eggs/g feces). Heavy *S. mansoni* infections were found to be associated with increased baseline risk of anemia, although children with moderate or heavy intensity infections had lower risk of physical wasting. Prevalence of egg-positive infection in the combined *S. haematobium* cohorts was 27%, with 5% of individuals having heavy infection (≥50 eggs/10 mL urine). At baseline, light intensity *S. haematobium* infection was associated with anemia and with lower scores in the social domain of health-related quality-of-life (HRQoL) assessed by Pediatric Quality of Life Inventory.

**Conclusions:**

Our consensus on practical markers of *Schistosoma*-associated morbidity indicated that height, weight, hemoglobin, exercise tolerance, HRQoL, and ultrasound abnormalities could be used as reference points for gauging treatment impact. Data collected over five years of program implementation will provide guidance for future evaluation of morbidity control in areas endemic for schistosomiasis.

**Trial registration:**

These cohort studies are registered and performed in conjunction with the International Standard Randomised Controlled Trial Registry trials ISRCTN16755535, ISRCTN14117624, ISRCTN95819193, and ISRCTN32045736.

**Electronic supplementary material:**

The online version of this article (10.1186/s12879-017-2738-5) contains supplementary material, which is available to authorized users.

## Background

Human schistosomiasis, also known as bilharziasis, is a tropical and sub-tropical disease that affects over 240 million people worldwide, with most infections occurring in sub-Saharan Africa [1–3]. *Schistosoma* infections account for at least 3.3 million disability-adjusted life years (DALYs) [3, 4]. Active infection is found most often among school age children [5–8], who acquire it through contact with fresh water containing infected snails. In Africa, morbidity is primarily caused by immunologic reactions to eggs that are produced either by *S. mansoni* worms inhabiting the blood vessels of the intestines or by *S. haematobium* worms inhabiting vessels of the genitourinary tract [1].

At present, the primary means of schistosomiasis control is mass drug administration (MDA) with the drug praziquantel. Current WHO guidelines on how to conduct MDA for control of severe morbidity were developed in 2002 and 2006 [9, 10] and were based on expert opinion. Because systematic evidence on the effectiveness of alternative multi-year regimens is needed to better define optimal approaches to control, the Bill and Melinda Gates Foundation funded the Schistosomiasis Consortium for Operational Research and Evaluation (SCORE) in 2008, through a grant to the University of Georgia Research Foundation [11, 12]. SCORE’s vision is to answer strategic questions to inform efforts to gain control of schistosomiasis in high-prevalence areas, to sustain control and move towards elimination in areas of moderate prevalence, and ultimately to eliminate *Schistosoma* transmission in at-risk communities.

Current WHO guidelines for control of *Schistosoma* species infections recommend an initial focus on prevention of infection-associated morbidities through regular annual or biennial MDA to limit cumulative damage from infection and any subsequent reinfection [10]. The morbidities associated with schistosomiasis include anemia [3, 13–15], growth impairment [16–18], reduced memory, learning [19], and fitness [3], along with chronic pain [3], chronic inflammation [14], and focal organ damage and scarring of the colon and liver (*S. mansoni*) or of the genitourinary organs (*S. haematobium*) [1]. A major effort of the SCORE Project has been to perform multi-arm, multi-year, randomized intervention trials that evaluate the effect of timing and alternative approaches to MDA on changes in prevalence and intensity of schistosomiasis in affected African countries [12]. Because these studies also afforded an opportunity to investigate changes in morbidity following MDA, nested cohort studies of changes in schistosomiasis-associated morbidity were instituted within the SCORE intervention trials. This paper describes the decision-making process regarding which markers to study, and the subsequent design and implementation of prospective cohort studies. The results are expected to inform public health decision-making and design of neglected tropical disease programs, and provide data for cost-effectiveness analysis of different schistosomiasis control strategies evaluated in SCORE operational research trials.

## Methods

### Study design

**Fig. 1:**
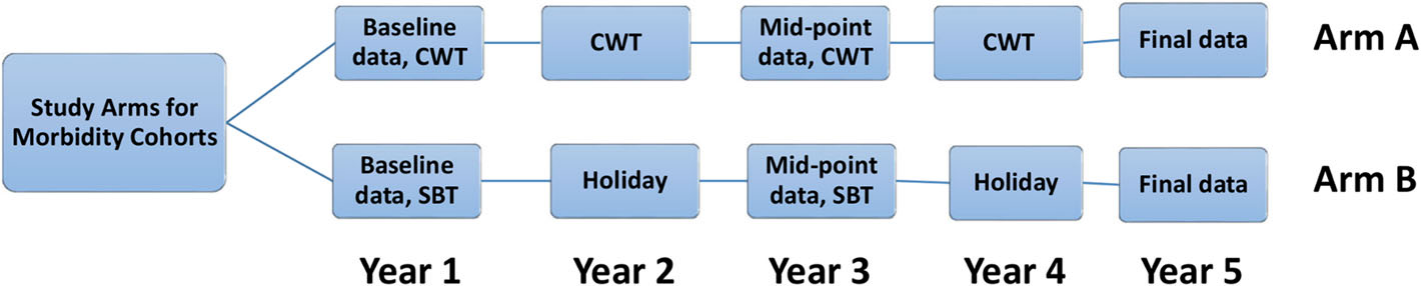
Diagram of the planned study arms for the SCORE cohort studies. As part of the larger SCORE randomized trials studying MDA delivery options for communities with endemic *S. mansoni* or *S. haematobium* infections [12], seven and eight year-old children residing in villages randomized into each of two SCORE project study arms were enrolled in prospective cohort studies of *Schistosoma* infection-related morbidities. In Arm A, communities are to receive yearly community-wide MDA. In Arm B, treatment will be given to school age children every other year, with drug ‘holidays’ in Year 2 and Year 4. The present paper reports on baseline pre-treatment data from Year 1; follow up examinations are planned and will be reported for Year 3 and Year 5 in later publications

To evaluate changes in morbidity associated with MDA over 5 years, we have initiated a set of parallel prospective cohort studies of children between the ages of 7 and 8 years enrolled in the SCORE intervention trials in African country regions with high prevalence of infection (>25%) with either *S. mansoni* or *S. haematobium* [10]. In these cohort studies, we follow a group of randomly selected 7–8 year old children in villages randomly assigned to two different MDA intervention arms as part of SCORE ‘Gaining Control’ trials (ISRCTN16755535, ISRCTN14117624, ISRCTN95819193, and ISRCTN32045736). Cohort children have been enrolled from villages in the two arms of each study with divergent MDA intervention schedules: the arm delivering annual community-wide treatment (CWT, Arm A) and the arm delivering school-based treatment every other year (SBT, Arm B) (Fig. 1). To evaluate the effect of MDA on disease caused by *S. mansoni*, cohorts were enrolled in Kenya and Tanzania; to evaluate the effect of MDA on disease caused by *S. haematobium*, cohorts were enrolled in Mozambique and Niger. Morbidity in the selected cohort children is to be evaluated serially at baseline, and after 3 and 5 years.

Niger (in 2004) and Tanzania (in 2006) had previously implemented national or sub-national MDA programs, in collaboration with the Schistosomiasis Control Initiative, that included annual or every-other-year anti-schistosomal praziquantel treatment [20]. Although a number of communities in Kenya’s western Lake Victoria region had been screened and treated as part of prior *S. mansoni* research [21], Kenya did not have a national schistosomiasis control project at the outset of the current study. Likewise, Mozambique did not yet have a national control program at the beginning of the SCORE study.

### Study development: identifying morbidity measures for inclusion in the cohort study

In July 2009, SCORE leadership convened a meeting to discuss infection-associated morbidity markers as potential study outcomes in a study of the impact of alternative approaches to MDA. There was agreement that markers used in the SCORE morbidity studies should be feasible to perform in the field, as well as compelling to decision-makers, and that the morbidity-marker research should yield useful results in terms of program metrics for success. It was agreed that the optimal design for the current morbidity impact assessment was to create nested longitudinal cohorts of children who could be available at baseline, Year 3, and Year 5 for serial follow-up during the course of the larger SCORE MDA trials.

The *Schistosoma-*associated morbidity outcomes that were considered during the meeting and in follow-up discussions are listed in Table 1. Most of the measures considered assessed individual-level impact of MDA among children, but some measures, such as household income and reproductive health outcomes, indirectly assessed family or community-wide impacts. It was ultimately decided not to attempt to measure community-wide externalities [22], as these would be difficult to measure precisely, would take a longer time to manifest, and when detected, would be difficult to attribute specifically to the study interventions without concurrent collection of data in a number of untreated control communities.

**Table 1:** Metrics considered for inclusion in SCORE morbidity cohort studies

**Recommended for study, and included in the cohort evaluation protocol:**
*Anthropometric measures*: age-standardized height, weight, body mass index (BMI), mid-upper arm circumference (MUAC) [16–18]
Blood hemoglobin [3, 13–15]
Exercise tolerance measured by 20-m shuttle run (beep) test [23–26]
Health-related quality-of-life, measured by standardized PedsQL survey instrument [27, 28]
Ultrasonography of the abdomen and liver (*S. mansoni*) or of the kidneys and bladder (*S. haematobium),* using standardized protocols [29, 30]
**Considered, but not selected for study:**
*Individual-level outcomes*
Indicators of the mechanism for anemia: Zinc protoporphyrin, ferritin, hepcidin, C-reactive protein, Interleukin-6 [14, 31, 32]
Volitional activity (e.g., as measured by accelerometers, fitness trackers) [33, 34]
Attention span [19, 35]
School performance, school completion rates [19]
Serologic measures of collagen metabolism [36]
Urine abnormalities, e.g., albuminuria, hematuria [3, 37]
Eosinophilic cationic protein (ECP) in urine [38]
*Household or Population-level outcomes*
Changes in religious participation [39]
Health system utilization [40]
Household income, productivity, work yield [22]
Community fertility and birth outcomes [41, 42]
Vaccine responses in babies [43]

The two sections indicate those that were selected and those that were not selected for inclusion in the final protocol

Selected references for each considered outcome are cited in brackets after each listing

The criteria for marker selection included: i) the likelihood that findings would be persuasive to program managers and policy-makers; ii) the existence of prior evidence suggesting a relationship between *Schistosoma* infection and its incidence; iii) the likelihood that the marker could be reliably measured in areas with limited infrastructure; iv) the cost of testing; and v) the potential for the marker to change during the period of study observation.

In addition to the outcomes included in the baseline dataset, data on mid-upper arm circumference (MUAC) were requested in the final protocol. However, as these MUAC data were incomplete and inconsistent, they were dropped from the final analysis.

### Selection of the cohort populations

The recruitment goal of the cohort study was to enroll 800 subjects from each of the four participating countries—400 from Arm A (receiving the most intensive MDA by CWT) and 400 from Arm B (receiving less intensive MDA via every other year by SBT) (Fig. 1). The selection for study inclusion was conducted in a two-step process – the first step involving village selection and the second focused on the individual children within those villages.

The large intervention trials in which the cohorts were nested include 25 villages per arm. Four villages from the most- and the less-intensive intervention arms were randomly selected for inclusion in the cohort study. However, investigators were permitted to select from a restricted group of no less than 10 of the 25 villages in each arm, which could be chosen, for example, based on ease of access from the study base. In Kenya, because fewer than 800 children were enrolled in the eight selected villages, two additional villages were added to each arm, yielding a total of twelve cohort study villages in the western Kenya site.

Children in the selected villages were eligible for the cohort study if they were age 7 or 8 at the start of the study, attended a local school, and did not have a disability that precluded participation in all of the study health measurements, in particular, the 20-m shuttle run fitness test [23, 44]. If more than 100 children were eligible, participants were to be randomly selected.

### Baseline and follow-up evaluation of cohort children

At the time of enrollment into a cohort, each child underwent a standard evaluation for *Schistosoma* infection, which consisted of either stool [45] or urine [46] examination (see below), depending on whether the area was endemic for *S. mansoni* or *S. haematobium*. Demographic and anthropometric data were collected through a standardized history and examination that included height and weight, measured using standardized techniques (stadiometers and calibrated scales) [44, 47]. WHO Anthro software (version 3.2.2) was used to calculate age specific Z scores for height and weight. Significant wasting was defined as a body mass index (BMI)-for-age Z-score of < −2, and growth stunting was defined as a height-for-age Z-score of < −2. Symptom burden and well-being were assessed using PedsQL, a validated instrument with multiple domains for measuring health-related quality of life (HRQoL) in children [27, 48].

Kenya and Tanzania used two different versions of the PedsQL survey. Kenya used a longer 23-question version than did Tanzania, which used the 16-question version with 15 of them adopted but the last question left out due to irrelevance [24]. To check the validity of combining the PedsQL scores from the two *S. mansoni* countries, we abstracted a subset of Kenya questions that were the same as Tanzania’s and performed a sensitivity comparison. The Cronbach’s alpha between the long version and short version of Kenya PedsQL scores was 0.93, reflecting an excellent level of internal consistency. Both Niger and Mozambique used the long version of PedsQL having 23 items.

To assess aerobic fitness, all children performed a 20-m shuttle run per standard protocol [23, 25]. The shuttle run data were then converted to an estimated maximal oxygen consumption (VO_2_ max) [23], a measure of the maximum volume of oxygen that a subject can utilize during exertion. The typical normal range of VO_2_ max for healthy 8 year old schoolchildren is 43–60 mL/kg/min [26]. Hemoglobin was measured using capillary blood; anemia was defined as hemoglobin <11.5 g/dl for children residing below 1000 m altitude (Niger and Mozambique, and <11.7 for children residing at the ~1200 m altitude near Lake Victoria (Kenya and Tanzania) [49]. Abdominal and urographic ultrasounds were performed on cohort children according to WHO Niamey protocols [29]. In *S. mansoni*-endemic communities, liver texture patterns were graded from A-F, and those graded C–F were considered abnormal. Portal vein dilation was scored based on subject height-specific standards found in the protocol [29]. Normal main portal vein width values are <7 mm for a 90 cm tall child, and <12 mm for a 170 cm child. Because the baseline ultrasound data collected in Mozambique were lost, the results of urogenital ultrasounds are not reported in our combined analysis.

### Laboratory methods

Stool and urine specimens were collected from children and evaluated for presence of *Schistosoma* eggs. At *S. mansoni* study sites*,* stool samples were collected on three consecutive days from each child; two slides containing standardized smears prepared from each stool were examined by an experienced technician using the Kato-Katz method [45] and the eggs enumerated. For *S. haematobium,* two 10 ml aliquots from a single mid-day urine specimen were filtered [46] and the eggs counted by two independent, experienced technicians. A subject was deemed positive for *S. mansoni* infection if eggs were detected in at least one of the six Kato-Katz slides, and a subject was deemed positive for *S. haematobium* infection if at least one urine aliquot tested positive for eggs by filtration.

Intensity of infection was measured by counting numbers of eggs observed in each of six Kato-Katz samples for *S. mansoni* and in each of the two 10 mL filtered urine samples for *S. haematobium*. For *S. mansoni*, the actual egg counts were converted to eggs per gram (epg) by simple multiplication times 24 [45]. Egg counts for *S. haematobium* are given as eggs per 10 mL of urine (ep10mL). Based on the WHO guidelines [9], intensities of infections were categorized as light (1–99 epg), moderate (100–399 epg), or heavy (≥400 epg) for *S. mansoni* infections, and as light (1–49 ep10mL) and heavy (≥ 50 ep10mL) for *S. haematobium* infections.

### Statistical methods

For purposes of presentation of baseline cohort characteristics in this paper, children from the two *S. mansoni* sites (Tanzania and Kenya) were combined into one cohort dataset, and children from the two *S. haematobium* sites (Mozambique and Niger) were combined into a second cohort dataset. As planned in the cohort study design, all main analyses were performed separately on *S. mansoni* and *S. haematobium* datasets due to the significant biological differences between *S. mansoni* and *S. haematobium* infection-related pathologies.

Simple univariate and bivariate analyses were used to describe study participants’ demographics. Chi-square tests and t-tests were performed to compare results from children who were egg-positive with those who were not. Linear or generalized linear mixed models that adjusted for over-dispersion and clustering effect at the village level were used to describe patterns of morbidity markers as a function of predictors of interest. Predictors used in our multivariable models were chosen mainly based on expert opinion and adjusted for common factors such as age and gender. Models for VO_2_ max were only adjusted for sex, but not age, because age is used to calculate VO_2_ max from the 20-m shuttle run results. To evaluate for the potential effects of missing outcomes data, multiple imputation for missing values was implemented as a sensitivity analysis [50, 51]. All analyses initially conducted for available cases were later repeated on datasets with missing data being imputed multiple times, from which new statistical inference results were made, and these were compared with those generated from the original dataset. Data analyses were performed using SAS version 9.4 (SAS Institute Inc., Cary, NC). An α = 0.05 level was used for significance of all statistical tests and for the confidence interval calculations.

The morbidity markers in the cohort study were considered secondary outcomes of the cross-sectional studies, which were powered to detect the impact of alternative approaches to MDA treatment on measures of infection. Because the expected treatment impact on infection status per intervention arm was unknown, power analysis for the cohort studies’ morbidity markers was not done. However, to maximize chances of establishing specific intervention-related effects, we enrolled the largest sample possible given the available resources.

### Ethics statement: human subjects participation

Human subjects participated under protocols reviewed and supervised by institutional review panels in each African country and by their academic partners. The University of Georgia (UGA) Institutional Review Board conducted an administrative review of each cohort study to ensure that all individual panel reviews met UGA human subjects protection requirements. Written informed consent was obtained from parents of children in the study, and assent was obtained from participating children.

## Results

The SCORE morbidity combined cohorts had the following baseline characteristics: 2443 children from 36 villages in 4 countries were enrolled in the cohort at baseline – 1373 in the combined *S. mansoni* group and 1070 in the combined *S. haematobium* group. The four countries: Kenya, Tanzania, Niger, and Mozambique, enrolled 801, 572, 799, and 271 subjects, respectively. Baseline sample characteristics for each individual country are listed in Additional file 1: Tables S1-S4. Enrollment targets for participation were reached in Kenya and Niger, but not in Tanzania, while in Mozambique, inadvertent enrollment of children whose ages were not in cohort range resulted in the subsequent exclusion of 361 participants.

### Cohort baseline characteristics

#### Combined *S. mansoni* cohorts

Children in the combined *S. mansoni* cohort (Tanzania and Kenya) had an average age at enrollment of 7.5 years, and 53% were female (Table 2). Sixty-two percent of the children were overtly infected with *S. mansoni (*egg positive), with 33%, 20%, and 10% of the total *S. mansoni* cohort populations having light, moderate, or heavy infection intensities, respectively.

**Table 2:** Participant characteristics for the combined cohorts

Characteristics	*S. mansoni*		*S. haematobium*	
	*N* = 1373		*N* = 1070	
	n	%	n	%
Schistosome egg-positive children	843	62%^†^	281	27%
Female	721	53%	524	49%
Infection intensity of egg-positive children				
Light (1–99 epg; 1–49 eggs/10 mL)	439	33%	231	22%
Moderate^a^ (100–399 epg)	264	20%	NA^a^	NA^a^
Heavy (≥ 400 epg; ≥50 eggs/ 10 mL)	140	10%	50	5%
Stunting	88	7%	232	22%
Wasting	262	20%	123	12%
Anemia	549	42%	513	50%
Liver Pattern				
A	1120	86%	–	
B	174	13%	–	
C and above	7	<1%	–	
	mean	SD	mean	SD
Age (years)	7.5	0.5	7.5	0.5
VO2 (mL/kg/min)	49.0	3.2	50.2	3.4
Portal vein diameter (mm)	7.2	1.1	–	
PedsQL Total	84.6	12.7	86.6	19.9
Physical	89.1	16.8	87.4	24.1
Emotional	74.5	15.7	92.9	14.5
Social	91.6	16.5	84.0	27.2
School	80.5	17.5	85.0	15.9

^†^Not equal to the sum of light, moderate, and heavy infection prevalence due to rounding

^a^ *S. haematobium* intensity is classified as light or heavy; there is no intermediate intensity

Prevalence of stunting and wasting were 7% and 20%, respectively, among children in the *S. mansoni* cohort, and, overall, 36% of the *S. mansoni* group children were anemic. All but 5% of children completed liver ultrasound examinations. Of those with ultrasound data, 86% had liver pattern A, 13% had liver pattern B, and <1% had liver pattern C or higher. Among all the enrolled subjects, measured mean portal vein diameter was 7.2 mm and mean VO_2_ max was 49.0 ml/kg/min. Mean total score on the PedsQL HRQoL assessment was 85 out of 100, with the means of its four subdomains (physical, emotional, social, and school) being 89, 75, 92, and 81, respectively.

#### Combined *S. haematobium* cohorts

For the combined data from the *S. haematobium* country cohorts (Mozambique and Niger), mean age at enrollment was 7.5 years, and 49% of the subjects were female (Table 2). Prevalence of egg-positive *S. haematobium* infection across these two cohorts was 27%, with 22% and 5% of the total population having light and heavy infection intensities, respectively. Overall prevalence of stunting was 22%, and 12% were wasted. Fifty percent of the *S. haematobium* group children (egg-positive and egg-negative) were anemic. Among all the enrolled subjects, mean VO_2_ max was 50.2 ml/kg/min. The mean total PedsQL score was 87, with the means of subdomains (physical, emotional, social, and school) at 87, 93, 84, and 85, respectively.

### Univariate associations with infection

There were no significant differences between egg-negative and egg-positive children in anthropometric measures or shuttle run performance among children enrolled in either the *S. mansoni* or *S. haematobium* combined cohorts. There were no significant differences in liver ultrasound scores in the combined *S. mansoni* cohort. Among children in the *S. mansoni* combined cohort, only the emotional subdomain of PedsQL score and hemoglobin were found to be significantly different between egg-positive and egg-negative groups, with *S. mansoni* egg-positive children having a lower mean PedsQL emotional sub-score (73.7 vs. 76.0, *P* = 0.01) and lower hemoglobin levels (11.8 g/dL vs. 12.1 g/dL, P = 0.01) (Table 3).

**Table 3:** Univariable relationships with egg-positive infection^a^

	*S. mansoni* countries					*S. haematobium* countries				
	*S. mansoni* egg-positive		*S. mansoni* egg-negative			*S. haematobium* egg-positive		*S. haematobium* egg-negative		
	*N* = 843		*N* = 507			*N* = 281		*N* = 771		
	n	%	n	%	*P* value	n	%	n	%	P value
Female	436	52%	275	54%	0.37	138	49%	377	49%	0.95
Stunting	56	7%	32	7%	0.90	67	21%	163	25%	0.22
Wasting	164	20%	95	20%	0.88	26	10%	96	13%	0.21
Anemia	**366**	**44%**	**182**	**38%**	**0.02**	**172**	**64%**	**331**	**44%**	**< 0.01**
	mean	SD	mean	SD	P value	mean	SD	mean	SD	P value
Age	7.5	0.5	7.5	0.5	0.20	7.5	0.5	7.5	0.5	0.25
VO2	49	3.2	49	3.1	0.62	50.1	3.7	50.2	3.3	0.81
Hemoglobin	**11.8**	**2.3**	**12.1**	**2.1**	**0.01**	**11.0**	**1.6**	**11.5**	**1.6**	**< 0.01**
PedsQL										
Total	84.3	12.9	85	12.5	0.33	**80.7**	**21.9**	**89.3**	**18.0**	**< 0.01**
Physical	89	16.1	89.1	18	0.99	**81.8**	**27.4**	**90.1**	**21.8**	**< 0.01**
Emotional	**73.7**	**15.9**	**76**	**15.5**	**0.01**	**89.4**	**17.4**	**94.4**	**13.0**	**< 0.01**
Social	91.9	16.4	91.3	16.8	0.54	**75.6**	**32.0**	**87.8**	**23.6**	**< 0.01**
School	79.8	17	81.7	18.3	0.08	**78.2**	**15.1**	**87.4**	**15.4**	**< 0.01**

^a^Numbers in each category may be less than the total number of children in each cohort because of missing data. Bold font indicates statistically significant differences at the *P* < 0.05 level

In the combined *S. haematobium* cohort, egg-positive children had mean age that was similar to that of egg-negative children. Egg-positive children had a higher prevalence of anemia (64% vs. 44%, *P* < 0.01) and lower hemoglobin levels (11.0 g/dL vs. 11.5 g/dL, P < 0.01) than egg-negative children. In addition, all PedsQL scores (Total score and physical, emotional, social, and school subscales) were significantly lower among *S. haematobium* egg-positive vs. egg-negative participants (Table 3).

### Multiply-adjusted models

In multivariable-adjusted models for the combined *S. mansoni* cohort, heavy intensity of *S. mansoni* infection was positively associated with anemia (Table 4). However, having moderate and heavy intensity of infection had apparent protective effects against wasting (Table 5). Models considering VO_2_ max, stunting, liver pattern, and total and all subdomain PedsQL scores did not indicate statistically significant associations between those outcomes and infection intensity. Summary results for all adjusted models performed for the combined *S. mansoni* cohorts can be found in Additional file 1: Table S5.

**Table 4:** Multivariable models for anemia in *S. mansoni* cohorts^a^

Predictors	Adjusted OR	95% CI	Unadjusted OR	95% CI
*S. mansoni* Egg-negative	Reference	Reference	Reference	Reference
Light intensity	1.05	(0.79, 1.40)	1.05	(0.79, 1.40)
Moderate intensity	1.38	(0.98, 1.93)	1.37	(0.98, 1.93)
Heavy intensity	**1.78**	**(1.16, 2.72)**	**1.78**	**(1.16, 2.72)**
Age in years	0.90	(0.71, 1.14)	–	–
Female	0.95	(0.76, 1.20)	–	–

^a^Bold font indicates statistically significant differences at the P < 0.05 level

**Table 5:** Multivariable models for wasting in *S. mansoni* cohorts^a^

Predictors	Adjusted OR	95% CI	Unadjusted OR	95% CI
*S. mansoni* Egg-negative	Reference	Reference	Reference	Reference
Light intensity	0.87	(0.60, 1.27)	0.88	(0.61, 1.28)
Moderate intensity	**0.45**	**(0.28, 0.73)**	**0.45**	**(0.28, 0.72)**
Heavy intensity	**0.38**	**(0.21, 0.71)**	**0.37**	**(0.20, 0.69)**
Age in years	0.89	(0.65, 1.20)	–	–
Female	0.82	(0.61, 1.11)	–	–

^a^Bold font indicates statistically significant differences at the P < 0.05 level

In the combined *S. haematobium* cohort, light intensity of *S. haematobium* infection was positively associated with anemia. The adjusted odds ratio for those with heavy intensity infection was similar in magnitude, but it did not achieve statistical significance (Table 6). Models considering VO_2_ max, stunting, wasting, and total and physical, emotional, and school subdomain PedsQL scores did not yield statistically significant associations with infection intensity. Summary results for all adjusted models performed for the combined *S. haematobium* cohorts are presented in Additional file 1: Table S6.

**Table 6:** Multivariable models for anemia in *S. haematobium* cohorts^a^

Predictors	Adjusted OR	95% CI	Unadjusted OR	95% CI
S. haematobium Egg-negative	Reference	Reference	Reference	Reference
Light intensity	**1.70**	**(1.18, 2.45)**	**1.74**	**(1.21, 2.51)**
Heavy intensity	1.64	(0.86, 3.16)	1.68	(0.87, 3.22)
Age in years	**0.73**	**(0.56, 0.96)**	–	–
Female	0.88	(0.68, 1.14)	–	–

^a^Bold font indicates statistically significant differences at the P < 0.05 level

### Sensitivity analysis for effects of missing data using multiple imputation

All of the analyses above were based on inclusion of only those cases having complete data. As a sensitivity check, the potential impact of missing data was investigated through multiple imputation using the fully conditional specification (FCS) approach [52, 53], after which we repeated our analyses on the imputed datasets. The results from the imputed datasets did not differ significantly, and the changes in estimated effect sizes were negligible.

## Discussion

This paper describes the process we used for developing the cohort studies nested in the SCORE multi-country parallel randomized trials of drug-based control of schistosomiasis in sub-Saharan Africa, and provides the baseline data. These cohort studies serve as pragmatic comparative effectiveness trials, in that they take advantage of a unique opportunity to explore effects of two very different approaches to multi-year MDA on morbidity associated with either *S. mansoni* or *S. haematobium* infection among children. The present study asks the question: does annual community-wide treatment with praziquantel result in incremental health benefits in terms of physical growth and development, exercise tolerance, hemoglobin levels and anemia, liver ultrasound findings, and health-related quality-of-life, in comparison to every-other-year treatment administered through a school-based program?

The cohort design development targeted children aged 7 to 8 years old to maximize potential follow up participation throughout the five-year period of the project. Mozambique enrolled Standard 1 (First grade) students; however, because of students’ irregular school attendance, a significant proportion of these had ages outside the targeted 7–8 year old age range. As a consequence, the participation of children meeting protocol inclusion criteria in *S. haematobium* countries was lower than that in the *S. mansoni* cohort.

Prevalence of *Schistosoma* infection was lowest in Niger. The villages assigned to Arm A (4 years of CWT) had the lowest mean prevalence of all study arms in Niger [12]*.* Given that the purpose of the cohort study is to assess differences in morbidity markers between balanced study arms, the baseline Niger data are given in this paper, but because of potential trial outcomes bias caused by this imbalance, the Niger cohort was terminated after the baseline data collection.

Despite higher infection prevalence, the combined *S. mansoni* cohort had lower anemia prevalence than the combined *S. haematobium* cohort. The *S. mansoni* and *S. haematobium* cohorts also showed differences in the proportion of children who had growth stunting and wasting. The *S. mansoni* cohorts (Kenya and Tanzania) had a relatively low combined stunting rate and a moderate wasting rate, whereas in the *S. haematobium* cohort (Mozambique and Niger), the situation was reversed: stunting was more prevalent and wasting less prevalent in these locations affected by urogenital schistosomiasis. Growth stunting reflects long term undernutrition or chronic disease, while wasting implies somewhat normal linear growth followed by a more acute period of undernutrition that reduces the velocity of weight gain, or worse, causes weight loss, which affects the age-adjusted BMI Z-score [54].

In agreement with other studies, our baseline cohort data indicated significant associations between active (egg-positive) *Schistosoma* infection and hemoglobin/anemia in most of the univariate and all of the multivariate models. In contrast, our results suggesting that moderate and heavy intensity of *S. mansoni* infection had protective effects on wasting (Table 5) were unexpected. Additional explorations revealed that average weights were similar in subjects with different intensity level of *S. mansoni* infection, but cohort children who were moderately and heavily infected tended to be shorter. This could have led to a relative increase in the calculated BMIs in those participants (Additional file 1: Table S7).

In terms of study limitations, these cohort studies had relatively low sample size of children experiencing morbidity, and so they may not have the power to detect statistically significant differences between infected and egg-negative children at baseline. The numbers of villages enrolled for the cohort studies from each country varied from 7 to 12. Because village clustering effects were adjusted for in multivariate models, our power to detect statistically significant differences in those models was more limited [55, 56]. It is possible that there were unmeasured confounders that could have affected morbidity markers, e.g., the impact of concurrent malaria on anemia [56]. Some additional factors were measured in individual country cohorts, e.g., malaria was measured in Kenya and Niger [56], but it was not possible to include these factors across the combined-cohort analysis presented here.

A relative strength of these cohort studies is their ability to measure and monitor morbidity markers in a longitudinal fashion. Even if final prevalence and intensity differences between the comparison groups are smaller than anticipated, children in Arm A will be treated annually in contrast to treatment every other year in Arm B, and, therefore, Arm A children will potentially spend more time in an uninfected state, which should, in theory, reduce development of *Schistosoma-*associated morbidity.

In initiating these cohort studies, we have demonstrated the practicality and challenges of enrolling large numbers of children into longitudinal cohorts, and of monitoring multiple morbidity markers among children in field studies. Some of our sites, such as Mozambique, had relatively little parasite control infrastructure prior to the initiation of the SCORE studies. Nevertheless, they were able to complete these baseline studies. The loss of the baseline ultrasound data in Mozambique was unfortunate, but the examinations themselves were conducted without a problem. We look forward to the final outcomes of the cohort study and to further assessment of the utility of our morbidity markers in measuring the impact of different strategies used to deliver anti-schistosomal MDA. Continued follow up of the enrolled cohorts will allow better estimates of the interim (Year 3) and longer-term (Year 5) benefits of participation in more- or less-intensive standardized MDA programs (Arm A vs. Arm B). Analyses should be able to quantify the magnitude of MDA effects on infection intensity, as well as on infection-related organ damage, anemia, growth, fitness, and health-related quality-of-life. It is expected that this empiric evidence can help to guide future ‘preventive chemotherapy’ approaches [10] to schistosomiasis morbidity control.

## Conclusions

Our consensus on practical markers of *Schistosoma*-associated morbidity indicated that height, weight, hemoglobin, exercise tolerance, HRQoL, and ultrasound abnormalities could be used as reference points for gauging treatment impact. Data collected over five years of program implementation will provide guidance for future evaluation of morbidity control in areas endemic for schistosomiasis.

## Additional file

Additional file 1: Document file containing Tables S1-S7. (PDF 142 kb)

## Abbreviations

BMI: Body mass index
CWT: Community-wide treatment
ECP: Eosinophil cationic protein
EP10ML: *Schistosoma haematobium* eggs per 10 mL urine
EPG: *Schistosoma mansoni* eggs per gram feces
HRQoL: Health-related quality of life
ISRCT: International Standard Randomised Controlled Trial Registry
MDA: Mass drug administration
MUAC: Mid-upper arm circumference
PEDSQL: Pediatric quality of life inventory questionnaire and scale
SBT: School-based treatment
SCORE: Schistosomiasis Consortium for Operational Research and Evaluation
UGA: University of Georgia
VO_2_ max: Maximum volume of oxygen uptake during exercise in mL per kilogram body weight per minute
WHO: World Health Organization
