# Supplementary material for: Protocol and baseline data for a multi-year cohort study of the effects of different mass drug treatment approaches on functional morbidities from schistosomiasis in four African countries
Source: BMC Infect Dis. 2017 Sep 29;17:652. doi: 10.1186/s12879-017-2738-5 (PMC5622450; doi:10.1186/s12879-017-2738-5)
Supplement: Additional file 1: — Document file containing Tables S1-S7. (PDF 142 kb) [file 12879_2017_2738_MOESM1_ESM.pdf]

Additional File 1: SUPPLEMENTAL TABLES

Supplemental Table S1. Participant characteristics for Kenya (*S. mansoni*)

**Characteristics**	**N = 801**	
	**n**	**%**
Schistosoma mansoni egg-positive children	507	65%
Female	406	51%
Infection intensity of egg-positive children		
Light (1 – 99 epg)	249	32%
Moderate (100 – 399 epg)	173	22%
Heavy (≥ 400 epg)	85	11%
Stunting	72	9%
Wasting	216	29%
Anemia	334	45%
Liver Pattern		
A	561	76%
B	171	23%
C and beyond	3	<1%
	**mean**	**SD**
Age (years)	7.5	0.5
VO2 (mL/kg/min)	47.5	2.0
Portal vein diameter (mm)	7.4	1.1
PedsQL Total	85.9	4.6
Physical	92.0	6.6
Emotional	71.5	9.9
Social	96.0	8.1
School	80.3	12.7

Supplemental Table S2. Participant characteristics for Tanzania (*S. mansoni*)

**Characteristics**	**N = 572**	
	**n**	**%**
Schistosoma mansoni egg-positive children	336	59%
Female	315	55%
Infection intensity of egg-positive children		
Light (1 – 99 epg)	190	33%
Moderate (100 – 399 epg)	91	16%
Heavy (≥ 400 epg)	55	10%
Stunting	16	3%
Wasting	46	8%
Anemia	215	38%
Liver Pattern		
A	559	99%
B	3	< 1%
C and beyond	4	< 1%
	**mean**	**SD**
Age (years)	7.4	0.5
VO2 (mL/kg/min)	51.2	3.4
Portal vein diameter (mm)	6.9	0.9
PedsQL Total	82.3	19.7
Physical	84.2	25.5
Emotional	79.5	21.5
Social	84.3	23.1
School	80.9	23.4

Supplemental Table S3. Participant characteristics for Niger (*S. haematobium*)

**Characteristics**	**N = 799**	
	**n**	**%**
Schistosoma haematobium egg-positive children	170	21%
Female	402	50%
Infection intensity of egg-positive children		
Light (11 - 49 ep10mL)	148	19%
Heavy (≥50 ep 10mL)	22	3%
Stunting	177	22%
Wasting	108	14%
Anemia	362	45%
	**mean**	**SD**
Age (years)	7.5	0.5
VO2 (mL/kg/min)	50.1	3.2
PedsQL Total	96.2	5.4
Physical	99.1	4.1
Emotional	99.2	3.5
Social	97.1	6.9
School	89.7	10.3

Supplemental Table S4. Participant characteristics for Mozambique (*S. haematobium*)

**Characteristics**	**N = 271**	
	**n**	**%**
Schistosoma haematobium egg-positive children	111	44%
Female	122	45%
Infection intensity of egg-positive children		
Light (11 - 49 ep10mL)	83	33%
Heavy (≥50 ep 10mL)	28	11%
Stunting	55	22%
Wasting	15	6%
Anemia	151	63%
	**mean**	**SD**
Age (years)	7.6	0.5
VO2 (mL/kg/min)	50.4	4.2
PedsQL Total	50.9	11.5
Physical	43.9	16.5
Emotional	69.4	15.7
Social	35.3	17.2
School	62.3	18.5

Supplemental Table S5. Summary table for adjusted models of *S. mansoni* association with studied morbidity markers

**Morbidity markers**	**Adjusted OR (95% CI) for binary outcomes***			
	Egg-negative	Light intensity	Moderate intensity	Heavy intensity
Anemia	-	1.05 (0.79, 1.40)	1.38 (0.98, 1.93)	**1.78 (1.16, 2.72)**
Stunting	-	1.02 (0.57, 1.83)	0.59 (0.28, 1.24)	0.60 (0.24, 1.51)
Wasting	-	0.87 (0.60, 1.27)	**0.45 (0.28, 0.73)**	**0.38 (0.21, 0.71)**
	**Adjusted model coefficients (95% CI) for continuous outcomes***			
VO_2 max	-	-0.07 (-0.44, 0.30)	-0.28 (-0.73, 0.16)	-0.11 (-0.66, 0.45)
Portal vein diameter	-	-0.02 (-0.16, 0.12)	0.15 (-0.03, 0.32)	0.14 (-0.08, 0.36)
Total PedsQL	-	-0.57 (-1.80, 0.66)	0.21 (-1.29, 1.70)	-0.17 (-2.04, 1.69)
Physical PedsQL	-	-0.80 (-2.43, 0.84)	0.27 (-1.72, 2.26)	-0.45 (-2.93, 2.03)
Emotion PedsQL	-	-0.79 (-2.76, 1.19)	-0.74 (-3.14, 1.66)	-1.02 (-4.01, 1.97)
Social PedsQL	-	0.30 (-1.32, 1.92)	-0.01 (-1.98, 1.96)	0.12 (-2.33, 2.58)
School PedsQL	-	-0.46 (-2.56, 1.63)	0.39 (-2.15, 2.94)	0.90 (-2.27, 4.07)

* Models are adjusted for age and gender, except for VO_2 max, which was only adjusted for gender. Bold font indicates statistically significant differences at the $P < 0.05$ level.

Supplemental Table S6. Summary table for adjusted models of *S. haematobium* association with studied morbidity markers

Morbidity markers	Adjusted OR (95% CI) for binary outcomes*		
	Egg negative	Light intensity	Heavy intensity
Anemia	-	**1.70 (1.18, 2.45)**	1.64 (0.86, 3.16)
Stunting	-	1.12 (0.76, 1.65)	1.08 (0.53, 2.20)
Wasting	-	0.86 (0.52, 1.41)	0.51 (0.15, 1.69)
	Adjusted model coefficients (95% CI) for continuous outcomes*		
VO_2 max	-	-0.38 (-0.93, 0.17)	0.49 (-0.51, 1.49)
Total PedsQL	-	0.16 (-0.84, 1.15)	-0.17 (-1.99, 1.66)
Physical PedsQL	-	0.23 (-0.93, 1.38)	0.13 (-1.98, 2.24)
Emotion PedsQL	-	0.97 (-0.35, 2.29)	0.69 (-1.72, 3.10)
Social PedsQL	-	-1.06 (-2.55, 0.43)	-0.78 (-3.53, 1.96)
School PedsQL	-	0.22 (-1.65, 2.08)	-1.74 (-5.13, 1.65)

* Models are adjusted for age and gender, except for VO_2 max, which was only adjusted for gender. Bold font indicates statistically significant differences at the P < 0.05 level.

Supplemental Table S7. Children weights and heights from different intensity categories in *S. mansoni* countries

Mean (Median)	Egg-negative	Light intensity	Moderate intensity	Heavy intensity
Weight (kg)	21.5 (21.0)	21.1 (21.0)	21.0 (21.0)	21.5 (21.0)
Height (cm)	122.7 (121.0)	122.5 (122.0)	121.4 (120.5)	122.0 (120.75)
